# Advanced Biosensing Strategies for Last-Line Antibiotics Vancomycin, Colistin, Daptomycin and Meropenem: Comparative Analysis of Electrochemical and Optical Detection Methods

**DOI:** 10.3390/antibiotics15040327

**Published:** 2026-03-24

**Authors:** Vivian Garzon, Daniel G.-Pinacho, J.-Pablo Salvador, M.-Pilar Marco, Rosa-Helena Bustos

**Affiliations:** 1 Doctoral Programme of Biosciences, Universidad de La Sabana, Chía 140013, Cundinamarca, Colombia; viviangaru@unisabana.edu.co; 2 Evidence-Based Therapeutics Group, Department of Clinical Pharmacology, Faculty of Medicine, Universidad de La Sabana, Campus del Puente del Común, Km. 7, Autopista Norte de Bogotá, Chía 250001, Cundinamarca, Colombia; daniel.g.pinacho@gmail.com; 3 Centro de Investigación Biomédica en Red en el área Temática de Bioingeniería, Biomateriales y Nanomedicina (CIBER-BBN), 08034 Barcelona, Spain; jpablo.salvador@iqac.csic.es (J.-P.S.); pilar.marco@cid.csic.es (M.-P.M.); 4 Nanobiotechnology for Diagnostics (Nb4D), Department of Surfactants and Nanobiotechnology, Institute for Advanced Chemistry of Catalonia (IQAC), Spanish Council for Scientific Research (CSIC), 08034 Barcelona, Spain; 5 Evidence-Based Therapeutics Group, Clínica Universidad de La Sabana, Campus del Puente del Común, Km. 7, Autopista Norte de Bogotá, Chía 250001, Cundinamarca, Colombia

**Keywords:** therapeutic drug monitoring (TDM), antimicrobial, antimicrobial resistance (AMR), daptomycin, vancomycin, meropenem, colistin, antibodies, surface plasmon resonance-imaging (SPRi), electrochemical biosensor

## Abstract

**Background/Objectives**: In the area of pharmacology and clinical research, it is necessary to use versatile technologies able to quantify last-line antibiotic molecules with high specificity and sensitivity. This article describes the development of two types of immunosensors based on amperometric and surface plasmon resonance (SPR) measurements and their applicability in the measurement/assessment of therapeutic drug monitoring (TDM) of four last-line antibiotics such as vancomycin, colistin, daptomycin and meropenem in human plasma. In this study, ligand immobilization by preconcentration assays, sensor surface regeneration, determination of sensitivity and correlation of plasma sample quantification results by HPLC were considered. **Results**: In the case of the electrochemical biosensor the IC50 values obtained were 3.49 μg/L for vancomycin (VAN), 5.44 μg/L for colistin (COL), 0.82 μg/L for meropenem (MER) and 5.10 μg/L for daptomycin (DAP). For the SPRi biosensor the LODs achieved were 19 ng/mL for VAN, 9 μg/L for COL, 12 μg/L for MER and 12.3 μg/L for DAP. Finally, both electrochemical biosensor and the SPRi optical biosensor showed that for the four antibiotics the standard deviations were less than 10% with respect to the HPLC results, with ranges for VAN between ~5–6 µg/mL, for COL ~0.2–0.7 µg/mL, for MER ~4.5–5.5 µg/mL and for DAP ~0.09–0.65 µg/mL. **Conclusions**: These kinds of biosensors provide a precise and sensitive strategy, together with real-time determination, to quantify last-line antibiotics, with working ranges like those shown by robust techniques such as HPLC and great potential for the clinic.

## 1. Introduction

Antimicrobial resistance (AMR) has been increasing in recent decades, considered a major health threat to patients and healthcare systems worldwide, because infections with AMR generate treatment failures, which lead to serious pathologies, prolonged hospitalizations and high healthcare costs [1]. Technological advances have allowed the discovery of different types of antibiotics with the ability to inhibit the growth of pathogens that threaten the lives of patients [2]. However, as antibiotics have improved, bacteria have found ways to survive these drugs by acquiring different resistance mechanisms, whether intrinsic, acquired or transmissible [3]. Examples of these are *Haemophilus gonococcus* (beta-lactams), *Pseudomonas* (aminoglycosides), *Neisseria gonorrhoeae* (glycopeptides), *Campylobacter* (tetracyclines) among others [4]. The above situation has contributed to the use of last-line antibiotics, which have a narrow therapeutic range and high rates of toxicity [5].

To minimize the impact of antimicrobial resistance on patient health, the development of personalized medicine could be of great help, where the evolution of the patient and the concentrations of the drug in the body fluids are the reference for decision making regarding treatment and dosage [6]. Novel strategies are currently being implemented to monitor last-line antibiotics, to achieve a TDM of the patient and thus reduce the impact of antibiotic resistance. To this end, different strategies have been implemented that allow adequate and accurate monitoring of antibiotics, where chromatographic methods and immunoassays are the most used [7]. They are very sensitive and specific techniques but are also expensive and time consuming, requiring laboratories and specialized personnel, making them monitoring solutions not completely useful in clinics.

Nevertheless, a new class of devices is revolutionizing the way of measuring drug concentrations in body fluids, especially in blood, plasma, serum and urine, called biosensors [8]. These devices are used to monitor different medications in a simple, fast and low-cost way, with the advantage that they can be used at the patient’s bedside by doctors or health personnel. Currently, biosensors are an alternative to different traditional methods due to their high detectability and specificity, short analysis times, automation, real-time work and low costs [9]. Most biosensors have been applied mainly in the clinical field focused on diagnosis, although due to their versatility and technological advances their use has extended to the food, agricultural, pharmaceutical and industrial sectors [10]. However, despite its great advantages, its presence in the market has been reduced.

**Figure 1:**
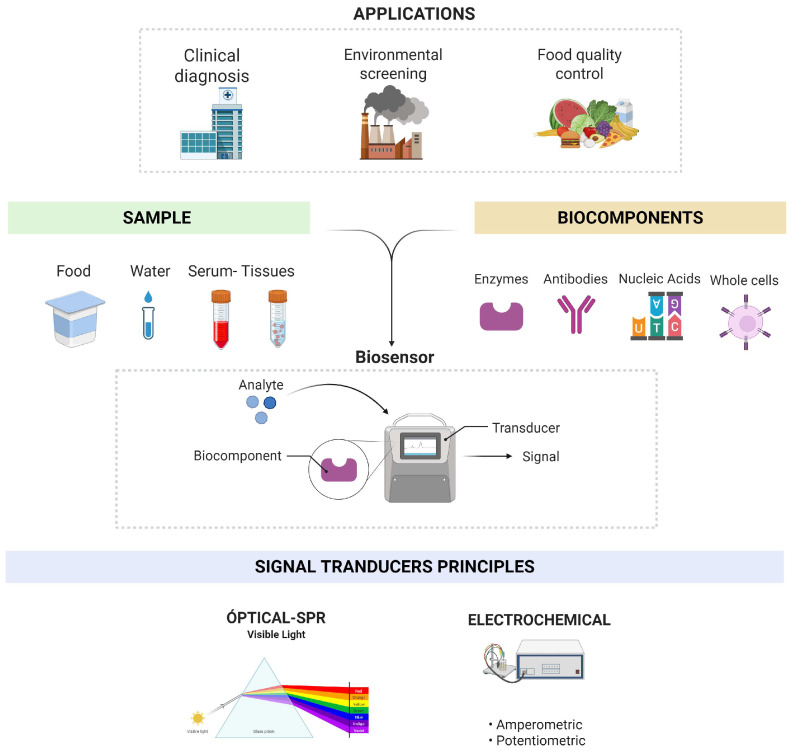
Uses of electrochemical and optical biosensors for the measurement of antibiotics and molecules of medical interest. Electrochemical biosensors have been used for the detection of antibiotics in different matrices, especially foods, and for the quantification of markers for cancer, pathogenic bacteria and drugs that require TDM.

Basically, biosensors are classified depending on the transducing principles that they are using which are mainly electrochemical and optical. Electrochemical biosensors monitor the electron transfer in the interface between the biofunctionalized electrode and the near environment. The amperometric biosensor, which is the most common one found in the literature, has been used for the detection of antibiotics in different matrices, especially foods such as milk, eggs and honey, among others, but there are few studies for human samples (Figure 1) [11]. The main antibiotics detected by this kind of sensors are those belonging to the antibiotic families of tetracyclines, sulfonamides, β-lactams and phenicols [11,12]. For example, penicillin G in human serum has been detected using polyclonal antibodies with an LOD of 0.003 ng/mL [13] and tobramycin in human serum has been quantified with a detection range of 2–6 µM [14]. Despite its widespread use and numerous advantages, it has some disadvantages, including potential electroactive interference from compounds present in the sample, such as proteins or organic acids, which can generate unwanted currents [15,16].

Optical biosensors have been used as an alternative in clinical diagnosis due to their ability to detect molecules in real time [17]. Surface plasmon resonance (SPR) biosensors are based on an optical measurement of refractive index changes using monochromatic light that excites plasmons. In other words, the SPR biosensor does not detect refracted light, but rather the change in the resonance angle, which manifests as a change in reflected light [18]. SPR optical biosensors have been used in various fields, primarily in food, water and other industries; however, there are not many reports in the literature on the quantification of antibiotics in clinical samples. Like electrochemical biosensors, the major drawback of this type of technique is the sample matrix, as it can interfere with the signal obtained [19].

For TDM, the quantification of amikacin using SPR is one of the most frequently reported techniques in the literature. Amikacin is reported in the literature as a last-line antibiotic that produces extremely serious side effects such as nephrotoxicity and ototoxicity. This antibiotic have been quantified by the use of optical biosensors, allowing rapid and sensitive quantification in plasma [20]. According to the literature, it has been quantified in human serum with an LOD of 0.13 ng/mL [17,20,21]. At the same time, chloramphenicol has been quantified in blood samples with a detection limit of 0.099 ± 0.023 ng/mL, lower than that reported by UPLC-UV [22].

This type of biosensor can also be used to quantify antineoplastic drugs that produce serious side effects that cause toxicity in patients.

Biosensors have advantages over other well-implemented analytical procedures such as high-performance liquid chromatography (HPLC) because of: (I) Low cost due to the reuse of sensor surfaces, not requiring highly qualified personnel, and low concentrations and volumes of reagents used; (II) Real-time responses because HPLC requires sample preparation and the equipment must be handled by trained personnel in a specialized laboratory; (III) Portability—it can be used at the patient’s bedside; and (IV) Automation—it can be fully automated for processing quickly in the laboratory [23]. This makes them a novel, useful, and very attractive tool for the quantification of antibiotic molecules in patient samples.

In this context, the objective of this study is to quantify clinically relevant last-line antibiotics with narrow therapeutic windows, namely vancomycin (glycopeptide), colistin (polymyxin), daptomycin (lipopeptide), and meropenem (carbapenem)—using two types of biosensors: electrochemical and optical. The methodology developed for plasma analysis was subsequently compared with a robust reference technique, high-performance liquid chromatography (HPLC). This approach aims to enable real-time quantification of last-line antibiotics, thereby facilitating therapeutic drug monitoring, dose optimization, and the early identification of potential treatment failure associated with bacterial resistance. To this end, polyclonal antibodies were generated in rabbits and characterized by ELISA [24]. Antibiotic concentrations were then determined using both biosensor platforms, and the results obtained from plasma samples were correlated with those generated by HPLC.

## 2. Results

### 2.1. Electrochemical Biosensor

**Figure 2:**
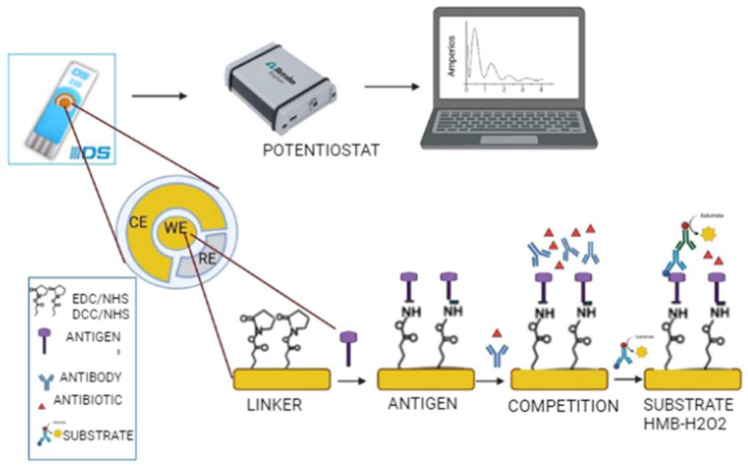
Schematic of the electrochemical biosensor for the detection and quantification of antibiotics. The protocol of the electrochemical biosensor consisted of biofunctionalizing the gold chips using the bioconjugates corresponding to each antibiotic, by means of activation via amino coupling, based on the covalent amino bond by previous activation of the carboxyl groups using NHS/EDC. After biofunctionalization, the competition between the primary antibodies/antiserum and antibiotic follows, then the addition of antiIgG-HRP and substrate to achieve the amperometric measurement. Differences between the signals obtained when the substrate solution (S) is added and the baseline signal recorded with the citrate buffer (C) were considered as the specific signal produced by the antiIgG bound to the bioconjugate.

An amperometric immunosensor was developed for monitoring VAN, COL, MER and DAP in human plasma. Amperometric measurements were recorded using a gold SPE biofunctionalized with the antigen. The biofunctionalization was achieved through the formation of self-assembled monolayers (SAM) (Figure 2).

#### 2.1.1. Regeneration Tests

**Figure 3:**
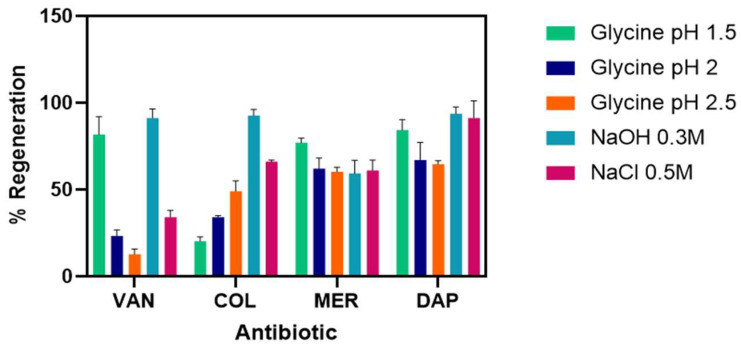
Agents used for the regeneration of the electrochemical biosensor chips. Values correspond to the baseline intensity after regeneration. Regeneration assays allow for chip reuse. During regeneration, the analyte must be removed, but the binding site must remain intact for new binding assays. Percentages above 80% were considered optimal for continuing analyte injection and ensuring assay consistency and reproducibility. Bars represent mean ± standard deviation (SD) from three independent measurements (*n* = 3) VAN: Vancomycin, COL: Colistin, MER: Meropenem, DAP: Daptomycin.

To reuse the electrodes after each measurement, different regeneration agents were tested. The objective was to break the interaction between the antigen and the primary antibody. These agents had to meet two important conditions: (i) keep the bioavailability of their ligand to ensure further binding to the analyte, and (ii) interact in a way avoiding damage to the sensor surface. Results of baseline intensity after regeneration are shown in Figure 3.

#### 2.1.2. Matrix Effect of Plasma on Amperometric Measurements

**Figure 4:**
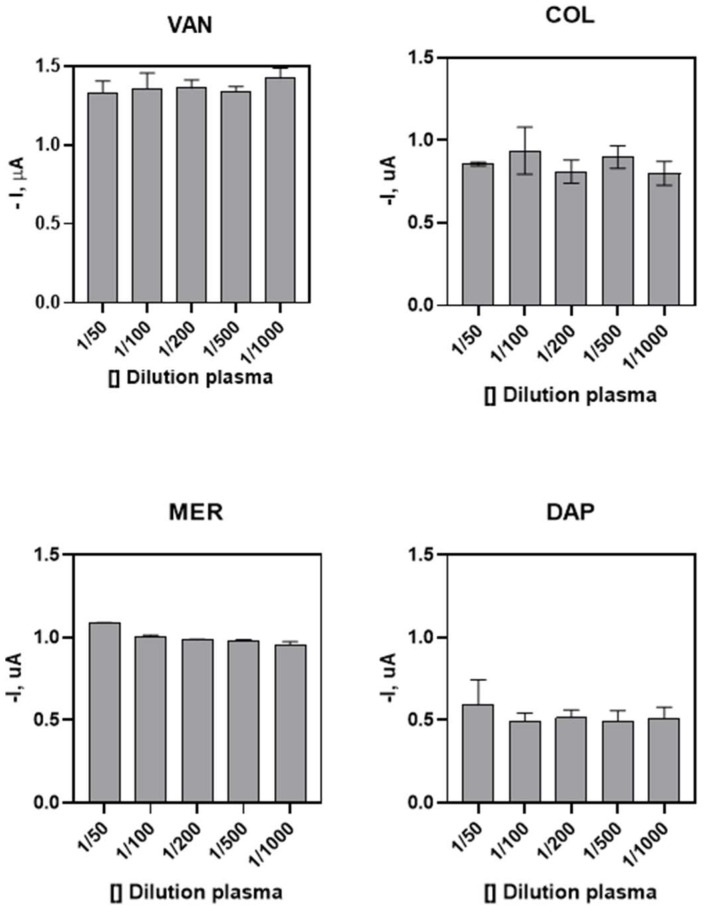
Matrix effect of plasma on amperometric measurements. Different dilutions of pure plasma (1/50–1/1000) with antiserum (1/10,000) belonging to each antibiotic were evaluated, with plasma diluted 1/100 to 1/1000 in PBST having a non-significant effect. -I, µA: current intensity microamps; []: concentration. Data are expressed as mean ± SD (*n* = 3 independent measurements).

To evaluate the matrix effect of plasma on biosensor measurements, different dilutions of pure plasma (1/50–1/1000) were evaluated using antiserum (1/10,000) corresponding to each antibiotic. For plasma diluted 1/100 to 1/1000 in PBST a non-significant effect was observed compared to the amperograms in PBST buffer solution (Figure 4).

#### 2.1.3. Calibration Curves

In quantification assays based on electrochemical biosensors, the analyte concentration is inversely proportional to the recorded current signal because the antibody competes with the antibiotic for a binding site. Plotting the logarithm of the concentration against the measured current (μA) yields a sigmoidal inhibition curve: a characteristic of the system was that increasing the analyte concentration progressively reduces the signal. This curve forms the basis for biosensor calibration and allows for the precise determination of the analyte concentration from the recorded electrochemical signals. The standard curves were fitted to a four-parameter equation according to the following formula: Y = [(A − B)/1 − (x/C)^D^] + B, where A is the maximum absorbance, B is the minimum absorbance, C is the concentration that produces 50% of the maximum absorbance, and D is the slope at the inflection point of the sigmoidal curve. From this equation, several characteristic parameters are defined, such as IC50 (analyte concentration corresponding to 50% inhibition), IC90/limit of detection (LOD) (analyte concentration corresponding to 10% inhibition), the linear working range where quantification is permitted between IC20 and IC80, and the slope, which indicates the assay sensitivity [25].

**Figure 5:**
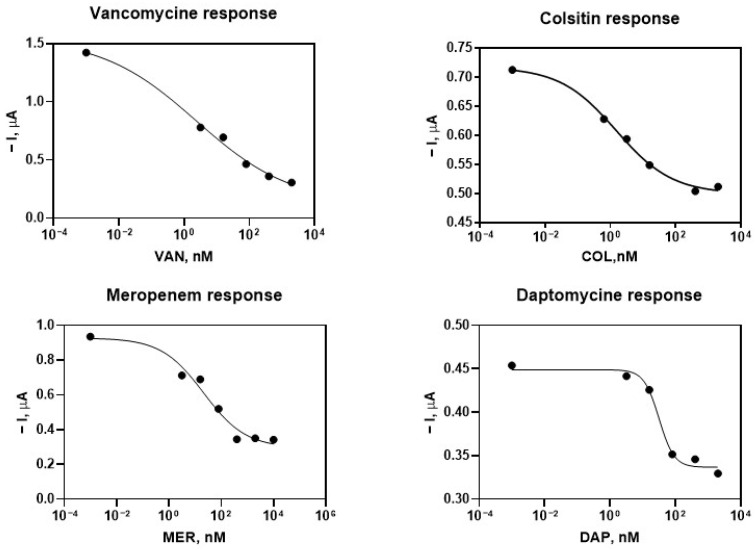
Calibration curves in spiked plasma diluted 1/100 for the antibiotics COL, VAN, DAP and MER, for the electrochemical biosensor. The standard curves were fitted to a four-parameter equation.

Calibration curves were carried out in 1/100 diluted plasma, finding that VAN has a very low IC50 of 2.41 nM, followed by MER with 21.41 nM, DAP with 31.52 nM and COL with 47.11 nM as seen in Table 1 and Figure 5.

### 2.2. Optical Measurements

The immobilization of antibodies on the sensor surface was done in three different steps: surface activation, coupling and regeneration. To complete the immobilization, it is necessary to select the appropriate pH for optimal binding. Three different pH values in acetate buffer were evaluated: pH 4.1, 4.5, and 5.5. pH 4.1 was selected because a higher peak in the sensogram was observed.

To determine that immobilization of the antibodies on the chip takes place, the amount of UR at the time of immobilization was considered, which is proportional to the surface concentration of the ligand [26].

#### 2.2.1. Regeneration Tests

**Figure 6:**
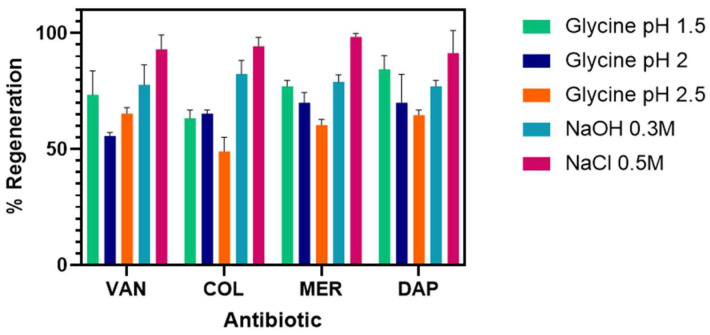
Sensory regeneration agent in SPRi. The percentage of regeneration achieved with the different regeneration agents. NaCl 0.5 M was chosen due to the high signal recovery obtained after treatment. All bar charts represent mean ± standard deviation (SD) from three independent measurements (*n* = 3).

Different regeneration agents were tested to find the one that minimizes damage and regenerates the sensor surface. Figure 6 shows the percentage of regeneration achieved with the different regeneration agents. NaCl 0.5 M was chosen due to the high signal recovery obtained after treatment for the chips biofunctionalized with the four antisera (Figure 6).

#### 2.2.2. Calibration Curves

Calibration curves were carried out in PBS buffer to verify the binding of the antibody to the antibiotic and as a control for comparison to determine the matrix effect of the plasma on the reflectivity units (UR).

**Figure 7:**
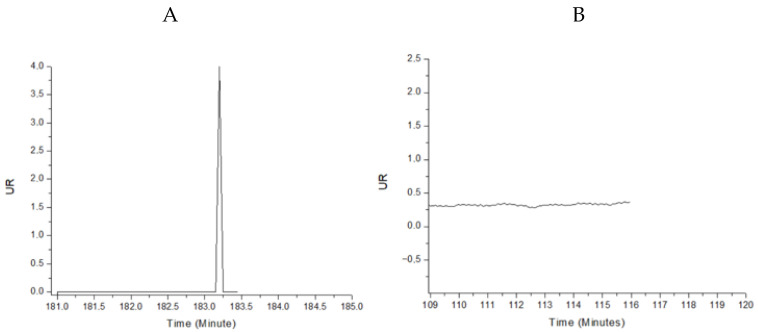
Plasma matrix effect. (**A**). Sensorgram of the untreated plasma sample. (**B**). Sensorgram of the treated plasma sample, protein precipitation with 50% (*v*/*v*) ethanol followed by 100-fold dilution and centrifugation for 15 min.

The matrix effect of plasma on biosensor measurements was first evaluated, for which different plasma dilutions were evaluated (1/50–1/1000). This demonstrated a matrix effect in plasma, with an injection signal observed at different plasma dilutions, with a change in RU relative to baseline. Due to the above, it was necessary to precipitate the plasma proteins that may be affecting the measurements [22]. Following protocols of the literature, protein precipitation with 50% (*v*/*v*) ethanol followed by 100-fold dilution and centrifugation for 15 min minimizes the matrix effect of plasma (Figure 7).

**Figure 8:**
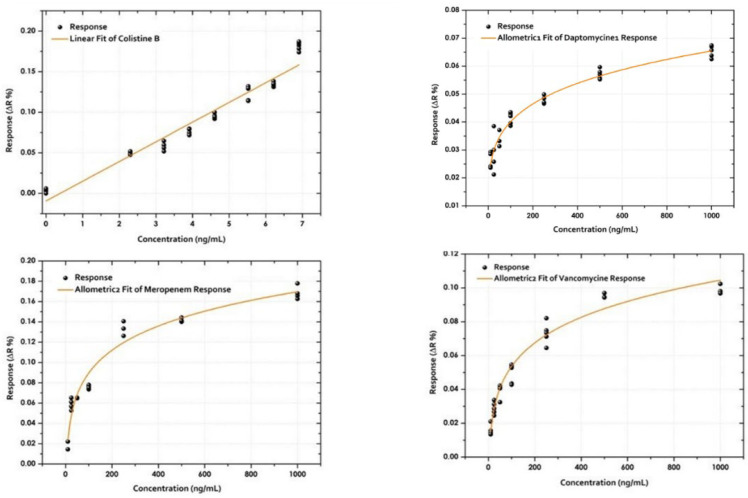
Calibration curves for VAN, COL, DAP and MER of the optical biosensor. The calibration curve was obtained by spiking the pure plasma with each of the antibiotics followed by treatment for protein precipitation. Each of the dilutions was then injected individually into the sensor surface corresponding to the specific antibiotic. The curve for COL had a linear fit and VAN, MER, DAP allometric fit.

Once the sample pretreatment was fixed, calibration curves were built (Figure 8).

Considering the calibration curves, the detection limits for each of the antibiotics are shown below (Table 2).

The limits of detection (LOD) are defined as the lowest concentration of ligand (antibody) that produces a response significantly greater than the mean response at 0 ng/mL determined by the following equation (LOD = 3 SD/S), where SD = Standard deviation of the response based on the standard deviation of the blank and S = Slope of the calibration curve. Likewise, the limit of quantification (LOQ) defined as the lowest concentration of ligand that could be quantified with high precision and accuracy was determined by the equation (LOQ = 10 SD/S).

Based on the above, it is important to highlight that the electrochemical biosensor has some advantages over SPRI in terms of cost and portability due to its size and the fact that the sample does not require processing. Conversely, SPRI has a lower turnaround time for obtaining results and requires a smaller sample volume (Table 3).

### 2.3. Surface Studies of the Chips

To determine the characteristics of the sensor surfaces, the electrochemical biosensor chips were observed using atomic force microscopy (AFM) checking the chip roughness (Table 4). On the other hand, for SEM it was evident that they had an organic content on the gold surface (darker shadows) in the form of filaments, in addition to crystals probably of salts coming from the running buffers (Figure S1).

The SPRi chips were observed, as well as those of the electrochemical biosensor, by atomic force microscopy (AFM). To determine whether there is a relationship between the immobilization of Ra: surface roughness, Rrms: total root means square roughness, Rmax: maximum height difference in the plane (Figure S1).

### 2.4. Comparison of Electrochemical and Optical Biosensor with HPLC

**Figure 9:**
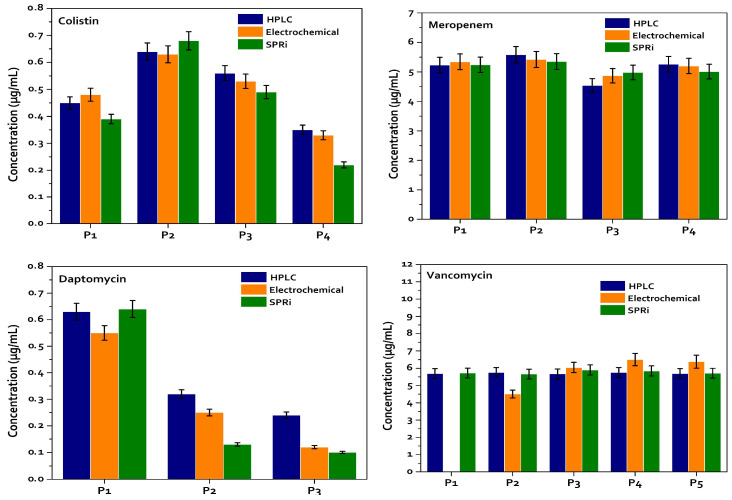
Comparison of HPLC, Electrochemical Biosensor and SPRi Optical Biosensor techniques. The data provided by the electrochemical biosensor and SPRi have a high similarity with the data provided by HPLC, the data between the optical biosensor and HPLC being closer. P: Patient. All bar charts represent mean ± standard deviation (SD) from three independent measurements (*n* = 3).

HPLC, electrochemical biosensor and SPRi optical biosensor techniques were compared by measuring the same patient samples using each technique. The data provided by the electrochemical biosensor and SPRi are very similar to the data provided by HPLC (Figure 9 and Table S1).

We performed a comprehensive statistical evaluation including Pearson correlation analysis, linear regression (R^2^), and Bland–Altman agreement analysis for each antibiotic (Figure S2).

Overall, the results indicate that agreement between methods is antibiotic-dependent. For colistin, a statistically significant and very strong correlation was observed between HPLC and the electrochemical biosensor (r = 0.978, *p* = 0.022), with a regression slope close to unity and a near-zero intercept, supporting good analytical agreement and practical interchangeability. Although SPRi also demonstrated very strong correlations (r > 0.98), the regression slopes (~1.5) revealed a proportional systematic bias, consistently yielding higher values; this was further reflected in the Bland–Altman plots, suggesting the need for a correction factor for direct equivalence. For daptomycin, very strong positive correlations were observed across all method comparisons (r > 0.96; R^2^ > 0.93). Despite marginal *p*-values likely attributable to the limited sample size (*n* = 3), the high correlation coefficients and slopes near unity (HPLC vs. electrochemical slope = 1.06) indicate robust linear agreement. As with colistin, SPRi comparisons showed proportional differences (slopes > 1), consistent with a systematic scaling effect rather than random disagreement. For meropenem, a strong and statistically significant correlation was observed between HPLC and the electrochemical platform (r = 0.957, *p* = 0.011), although the regression slope (0.54) indicated a scaling discrepancy. Comparisons involving SPRi showed moderate-to-strong correlations but without statistical significance, and Bland–Altman analysis suggested wider limits of agreement, indicating that calibration adjustments would be required for quantitative equivalence. Vancomycin demonstrated strong and statistically significant correlations (r ≥ 0.95; R^2^ ≈ 0.90) across the evaluated methods. The Bland–Altman analysis showed narrow limits of agreement relative to the mean concentrations, indicating a high degree of concordance. Collectively, these results support the analytical consistency and potential interchangeability of the methods for vancomycin quantification under the tested conditions. Taken together, these analyses provide a more rigorous assessment of analytical agreement.

## 3. Discussion

### 3.1. Electrochemical Measurements

For amperometric measurements, SAMs were used for biofunctionalization. SAMs are characterized by an organization of molecules at the solid–liquid interface induced by a strong chemisorption between the substrate and the head group [27]. For the formation of the SAM, MUA, a thiolated molecule that binds to the gold surface, was used, generating a covalent bond due to its high binding energy [28]. SAM formation was followed by activation of the carboxylic acids of MUA with EDC/NHS (100 µg/mL in PBS) or DCC/NHS (200 mM in DMF), covalent binding of the bioconjugates by forming an amide bond and finally blocking of the remaining carboxylic acids on the surface with ethanolamine.

To evaluate the effect of plasma matrix on biosensor measurements, different dilutions of pure plasma (1/50–1/1000) with antiserum (1/10,000) for each antibiotic were tested. Plasma diluted 1/100 to 1/1000 in PBST showed no significant effect when compared to amperograms in PBST buffer. This is possibly because the concentrations of the plasma components after the 1/100 dilution do not interfere with the current drops [29]. After determining the parameters involved in the measurements, calibration curves were generated.

Amperograms were acquired at an applied potential of −0.10 V versus the Ag reference electrode for 60 s. Signals were acquired at a flow rate of 100 µL/min. The recorded signal was the mean current value obtained during the last 20 s until steady state was reached. The difference between the signal obtained when the substrate solution was added and the signal recorded with citrate buffer was considered the specific signal produced by the antiIgG bound to the bioconjugate. Standard curves were fitted to a four-parameter equation.

Regeneration assays allow for chip reuse. During regeneration, the analyte must be removed, but the binding site must remain intact for new binding assays. For the regeneration assays, protocols previously described in the literature were followed [30,31], considering the physicochemical nature of the analyte.

The percentage of regenerated analyte obtained for each solution used was calculated using the following equation [1 − (Rreg/Ro) × 100], where Rreg was the RU after the regeneration solution was injected and Ro was the RU before the analyte injection [32]. Percentages above 80% were considered optimal for continuing analyte injection and ensuring assay consistency and reproducibility.

For each of the chips, different regeneration solutions were tested by evaluating the behavior of the amperograms, finding that for VAN and MER, Glycine pH 1.5 was the best agent of regeneration due to the starting signal was successfully recovered after each interaction (Figure 3). In addition, it is one of the most used regeneration agents for proteins of this size and is a low-cost alternative [33]. Since, by having both positive and negative charges, glycine is considered an agent of choice to separate molecules from the detection surface [26].

For COL chips, 0.3 M NaOH (10 min) followed by PBST buffer (5 min) shows the best performance as a regeneration agent (Table 2). Regeneration with NaOH is widely used to elute target molecules such as proteins from the detection interface [34], due to a sudden change in pH that allows the breaking of the interactions between the antibody and the bioconjugate [35]. In DAP chips, 0.5 M NaCl (10 min) was used as a regeneration agent; it is a very effective agent since it is a strong electrolyte that alters the ionic strength between the biofunctionalized protein and the antibody [33] (Figure 3).

After performing several cycles of measurements and regeneration at a 1/10,000 antibody dilution, the signal was similar in the five cycles evaluated. The results demonstrate that the chips of each bioconjugate can be reused for at least 5 cycles without any significant effect on the electrochemical response [36]. Vancomycin was an antibiotic that generated a greater signal, demonstrating that vancomycin antibodies can detect to a greater extent the bioconjugates immobilized on the chip.

The concentrations of plasma components after 1/100 dilution do not interfere in the maximum signal [29]. After determining the parameters involved in the measurements, calibration curves were carried out showing good detectability in plasma for the four antibiotics (Figure 5, Table 2), comparable to results previously published.

Regarding vancomycin, studies have been reported in the literature in which molecularly imprinted biosensors have been used for the selective detection of vancomycin in serum samples spiked with the antibiotic, with LOD of 0.002 nM [37]. At the same time, the use of graphene tube-modified electrodes has been reported for the detection of vancomycin in spiked plasma samples with LOD of 200 nM [38]. On the other hand, serum and urine samples spiked with vancomycin were passed over an electrode modified with a copper (II) benzene-1,3,5-tricarboxylate organometallic structure with poly (acrylic acid), finding LOD of 1 nM [39]. According to the above, it is possible to deduce that the electrochemical biosensors developed in this research have a high detectability, showing a plasma LOD close to those compared with other formats and modifications in the electrodes reported in the literature.

The analytical performance of both biosensing platforms was further characterized to support their clinical applicability for therapeutic drug monitoring. For each antibiotic, the limit of detection (LOD), limit of quantification (LOQ). Notably, the LOQs obtained for vancomycin, colistin, meropenem, and daptomycin were below the lower bounds of their respective therapeutic windows, ensuring reliable quantification at subtherapeutic concentrations. The linear ranges covered clinically relevant concentrations, encompassing subtherapeutic, therapeutic, and supratherapeutic levels. Accuracy was assessed by comparison with the reference HPLC method, demonstrating acceptable agreement within the therapeutic concentration intervals. Collectively, these results confirm that both the electrochemical and SPRi platforms meet essential analytical performance criteria and are suitable for clinically meaningful antibiotic quantification.

Studies have been carried out for the detection of colistin using electrochemical biosensors in different matrices, especially in foods such as chicken and its derivatives, by immobilizing antibodies on the surface of screen-printed electrodes [40]. However, in the reviewed literature there is no evidence of the quantification of this molecule in human fluids. On the contrary, it is generally used to evaluate the susceptibility of certain bacterial species using electrochemical biosensors in sputum and plasma [41]. This research is novel regarding the quantification of this type of antibiotic with a narrow therapeutic range for TDM using electrochemical biosensor.

For meropenem, a greater number of reports of quantification in human fluids are found in the literature. A study using pyrolytic graphite electrodes modified with carbon nanotubes for monitoring in human plasma is able to detect meropenem at 1500 nM [42]. Also, the voltammetric monitoring of meropenem in human plasma shows an LOD of 283 mM [43] and another study using electrodes modified with carbon nanotubes and hydroquinone reports an LOD of 1.3 nM [42]. The IC50 and LOD of the biosensor developed here are in most cases lower than those reported in the literature, making it a great alternative to measure meropenem in TDM.

Finally, for daptomycin there are few reports in the literature of quantification by electrochemical biosensors in human body fluids. One of these studies is based on the quantification of daptomycin using a molecularly imprinted nanosensor based on Au-Pt particles, in deproteinated human serum with an LOD of 0.00161 nM [44]. The LODs may be different from those found in this research, since these are different electrochemical assays with specific protocols for each technique, which allow the quantification of this type of molecule, with short sample pretreatment in our case. However, this research can be of great help when evaluating the concentration of VAN for TDM.

According to the results obtained, electrochemical biosensors are a great tool for the measurement of last-line antibiotics in patient plasma. They are easy-to-use and fast devices with real-time results that will allow health personnel to take the necessary measures to preserve the patient’s well-being. Compared to the literature, the electrochemical biosensors developed are novel and of great clinical importance for antibiotics such as colistin and daptomycin, since they have almost no reports of quantification in plasma and are molecules with narrow therapeutic ranges.

### 3.2. Optical Measurements

For the immobilization of antibodies on the surface it is important to select the appropriate pH to ensure optimal immobilization. For this purpose, 3 different pHs (4.1, 4.5 and 5.5) of the 10 mM sodium acetate buffer were tested, selecting the pH 4.1 buffer, because it presented a higher peak in the sensorgram as observed in Figure 6. For immobilization, pHs below the isoelectric point of the protein were used since they favor electrostatic interactions between positively charged proteins.

To determine if the immobilization of the antibodies on the chip takes place, the amount of UR at the time of immobilization was considered, finding that for the four antibiotics there was an increase in the surface concentration compared to the negative control. While comparing the antibiotics the UR are higher for DAP and VAN, while for COL and MER they have lower values close to 0.7 UR. This fact could be due to that the antibodies of DAP and VAN were in greater proportion in the antisera or possess a higher avidity for the antigen, generating a greater accumulation of mass which increases the reflection index [31].

For regeneration, 0.5 M NaCl was used since it had the highest regeneration percentage. NaCl is a widely used regeneration agent since, being a strong electrolyte, it can alter the ionic strength between the antibody and the antibiotic molecule, leaving the surface to be used again in the next assay [33]. This type of regeneration is an alternative to avoid irreversible denaturation of the biosensor components [45].

For calibration curves in PBS, a change in the UR for each of the antibiotics is evident as the antibiotic dilutions in 0.01 M PBS buffer increase. However, a matrix effect was evident in plasma. Due to the above, it was necessary to precipitate the plasma proteins that may be affecting the measurements [46]. Following the protocol described in the literature, matrix effect was minimized by protein precipitation with 50% (*v*/*v*) ethanol followed by 100-fold dilution and centrifugation for 15 min [47].

Compared to the literature, for VAN, no publications were found in which this antibiotic molecule is quantified by SPRi in human fluids. However, there are studies where vancomycin has been quantified in matrices such as milk with LOD of 4.1 ng/mL [48]. This opens the possibility to measure vancomycin in human fluids with LOD of 19 ng/mL. Regarding COL, research has been published based on the quantification of this antibiotic mainly in foods such as milk [49] as well as VAN. However, in human fluids, the quantification of polymyxin B-deoxycholate in human serum, a molecule similar to colistin, is reported [50]. Our study opens a way to quantify this type of highly toxic antibiotic in plasma in a simple and rapid manner with LOD of 9 ng/mL and LOQ of 21.3 ng/mL. The values were lower compared to the other antibiotics analyzed.

For DAP, no studies were found in which this type of antibiotic was quantified in plasma. However, due to the great clinical importance of this antibiotic and the structure-activity relationship, SPR was used to determine the binding profile of daptomycin and human serum albumin, opening a new possibility of modification of this type of molecule [50]. At the same time, this technique has been used to identify the binding of daptomycin to the membrane of Gram-positive, Gram-negative and mammalian bacteria [51]. For MER, as with the other molecules, no studies were found in the literature in which MER is quantified in human plasma using SPRi, but the use of this type of biosensor for the quantification of β-lactam antibiotics in foods such as milk [52] or in water samples [53] has been reported. According to the above, measurements of this type of molecule by an SPR biosensor have been carried out mainly to show contamination in foods such as milk, but they have not been widely used in clinical settings. Therefore, they are a new opportunity to carry out the measurement of last-line antibiotics in a simple and fast way, helping doctors to adjust the dose considering the plasma concentration.

Regarding the surface characterization of all functionalized chips of sensors, roughness increases with respect to the control due to the changes that the surface undergoes after immobilizing the antigens and performing the measurements in constant flow. This is since the SAM modifies the surface of the chip, and the buffers leave traces of salts on the surfaces, factors that increase roughness as shown in Table 4.

On the other hand, SEM showed that they had an organic part on the gold surface (darker shades) in the form of filaments, in addition to crystals probably of salts from the running buffers. Regarding the composition of atoms on the surface, gold predominates because the surface has a high percentage of this element. Followed by oxygen, typical of the pores of the sample; C, N, Na and P belonging to the proteins (antigen, antibody) and the buffers respectively. Also, it is possible to determine that the control chip showed the presence of C, O and Au and not of the other elements present when immobilizing the chips.

The results of the electrochemical biosensor and SPR are similar; however, the SPR biosensor required sample pretreatment because matrices containing molecules such as proteins or lipids can interfere with the refractive index [54]. Therefore, the sensor interprets these changes as signals, even though there is no actual biological binding. At the same time, nonspecific adsorption can occur, especially when the sample contains large molecules such as serum proteins, leading to surface saturation and loss of selectivity, resulting in surface fouling [54,55]. On the other hand, in electrochemical biosensors the signal depends on the redox reaction, so complex molecules such as proteins present in the samples do not directly alter the electronic transfer in the electrode [56].

### 3.3. Comparison of Electrochemical and Optical Biosensors with HPLC

Quantification of VAN, COL, MER and DAP was performed in samples from patients who are receiving antibiotics due to multi-resistant bacterial infection. It is important to note that this study was intended to make a preliminary comparison with a small number of patients, to compare the ability of the electrochemical biosensor and SPRi optical biosensor to quantify antibiotic molecules in the same range as a standard reference technique such as HPLC. The HPLC technique has been widely used in the literature as a reference to evaluate the application of both electrochemical and SPR biosensors used for TDM [57]. The HPLC technique is characterized by being robust, selective, sensitive and specific in the quantification of molecules in different matrices [58].

The HPLC has been used to quantify these types of antibiotics in patient samples, such as vancomycin in human plasma from critically ill patients with an LOD of 1 µg/mL [59]. Colistin in urine with an LOD of 100 nmol/L [60], and in plasma and kidney with a range between 50 ng/L and 150 ng/L [61]. Simultaneously, studies have been conducted on meropenem in human plasma with an LOD of 0.0018 µg/mL [62] and in cerebrospinal fluid with a range of 0.1 mg/mL to 100 mg/mL [63]. Daptomycin, on the other hand, has been quantified in plasma with an LOD of 1.56 µg/mL [64].

We compared the results obtained from the electrochemical biosensor with HPLC and found that for the four antibiotics the concentration ranges are close to each other with standard deviations less than 10%, which allows us to deduce that the data are close to each other. Figure 9 shows the concentration range of each of the antibiotics for the group of patients evaluated in the electrochemical biosensor for VAN, which ranges between ~4–10 µg/mL, COL ~0.4–0.7 µg/mL, MER ~4.5–6 µg/mL and DAP ~0.12–0.55 µg/mL. Therefore, these values suggest that the quantification of these antibiotic molecules by the electrochemical biosensor has a sensitivity similar to that of HPLC for quantifying VAN, COL, MER and DAP in plasma samples [58]. The above would be of great use in pharmacological terms since there are no reports in the literature on the quantification of these antibiotic molecules using this type of biosensor in patient plasma. Therefore, these results represent a great new opportunity for the quantification of last-line antibiotics at the patient’s bedside, considering the dimensions of the equipment and the protocol developed for this test, which may suggest that the data between the SPRi biosensor and HPLC are close to each other, as shown in Figure 9. These results, like those of the electrochemical biosensor, are a great advance in the quantification of antibiotics by SPRi since it is a technique that does not require organic solvents for the sample, making it a simple protocol for real-time quantification [57].

The range of concentrations in samples of patients quantified by the electrochemical biosensor and SPRi is below the therapeutic ranges of antibiotics. This may be due to the distribution of the antibiotic in the body (PK) [25] or due to the storage of the samples, since they were evaluated at different times after being collected and it is likely that the characteristics of the antibiotic molecules have been altered. There are no reports in the literature where the antibiotics VAN, COL, MER and DAP in plasma of patients are quantified and compared with HPLC using an electrochemical biosensor or SPRi, under the protocols established in this study. On the contrary, the protocol of this type of biosensor has been used for the quantification of disease markers such as cancer [57] and antibiotics in food [65]. This research represents a major step forward for personalized medicine in the search for solutions to combat the exponential growth of multi-resistant bacteria and their impact on individual and public health.

The stability of the biosensor platforms was evaluated at the operational level to assess short-term analytical robustness. Both the electrochemical and SPRi sensors demonstrated consistent signal responses over repeated measurements and regeneration cycles, maintaining signal recovery above 80% without significant loss of sensitivity. These results indicate acceptable short-term operational stability under the experimental conditions employed.

Plasma samples were processed and stored under controlled conditions consistent with previously validated HPLC protocols, ensuring sample integrity during comparative analysis. While comprehensive regulatory stability studies—such as extended long-term storage of functionalized sensor chips, inter-batch reproducibility assessment, and freeze–thaw stability under formal bioanalytical validation criteria—were beyond the scope of the present work, the observed reproducibility and regeneration performance support the analytical reliability of the proposed platforms. Future studies will be directed toward systematic long-term stability evaluation to further support clinical implementation and point-of-care applicability.

A potential limitation of the present study relates to the assessment of analytical selectivity in the context of structurally related antibiotics and commonly co-administered drugs. Although quantification was based on polyclonal antibodies specifically generated against vancomycin, colistin, meropenem, and daptomycin—and their sensitivity and selectivity have been previously characterized by our group, demonstrating high specificity and minimal cross-reactivity under controlled immunoassay conditions—additional interference studies were not performed within the framework of this work. Given that the primary objective was to compare the quantitative performance of the electrochemical and SPRi platforms with the reference HPLC method in human plasma, comprehensive cross-reactivity testing fell beyond the scope of the study. Nevertheless, the absence of systematic evaluation against structurally similar compounds or concomitant medications may limit the generalizability of the findings to more complex clinical scenarios, and dedicated interference studies should be incorporated in future validation efforts to further support clinical implementation.

## 4. Materials and Methods

### 4.1. Chemicals and Immunochemicals

Colistin (COL) and vancomycin (VAN) standards were supplied by Sigma-Aldrich (St. Louis, MO, USA). Daptomycin (DAP) (Cubicin^®^ 350 mg) was supplied by Novartis and meropenem (MER) by Farmacologíca S.A. 1-ethyl-3-(dimethylaminopropyl) carbodiimide hydrochloride (EDC), N-hydroxysuccinimide (NHS), dicyclohexylcarbodiimide (DCC), N, N-dimethylformamide (DMF) and 11-mercaptounecanoic acid (MUA) were supplied by Sigma-Aldrich (St. Louis, MO, USA). Acetone and Glycine 10 mM were supplied by Sigma-Aldrich (St. Louis, MO, USA). Sodium dodecyl sulfate (SDS) was supplied by Sigma-Aldrich (St. Louis, MO, USA). Secondary antibody antiIgG-HRP was supplied by Sigma-Aldrich (St. Louis, MO, USA). Plasma samples from the Red Cross blood bank were used for calibration curves.

The following immunoreagents, used for the development of the biosensors, were obtained as a result of previous research [24]. Coating antigens: VAN-DCC-BSA, COL-DMP-BSA, DAP-DCC-BSA, MER-DCC-BSA; antisera: vancomycin Rb 157, colistin Rb 66, meropenem Rb 161 and daptomycin Rb 156.

#### Buffer Solutions

Phosphate-buffered saline (PBS) was a solution of 0.01 M phosphate in 0.8% saline at pH 7.5. PBST was PBS with 0.05% Tween 20. For electrochemical measurements, substrate solution containing 0.001% TMB (3.3′,5.5′-tetramethylbenzidine) and 0.0004% H_2_O_2_ in 0.04 M citrate solution, pH 5.5, is used as measurement solution. Sodium hydroxide (NaOH) at different concentrations in 0.5 M sodium chloride (NaCl) was used as a regeneration solution. For SPRi measurements, 3 g/L sucrose was used as the calibration solution and 0.5 M NaCl as regeneration solution. The running buffer (measurement solution) for SPRi assays was in PBS. The equipment washing solutions were ultrafiltered water and 1% sodium dodecyl sulfate (SDS).

### 4.2. General Instruments

The pH and conductivity of all buffers and solutions were measured with a pH meter, pH 540 GLP, and a conductivity meter, LF 340, respectively (WTW, Weilheim, Germany). The peristaltic pump used was Miniplus^®^3 (GILSON, Middleton, WI, USA) with a Tygon LMT-55 orange/yellow tubing from Health and Science GmbH, Wertheim, Germany, with an ID of 0.75 mm and a wall of 0.91 mm.

#### 4.2.1. Electrochemical Instrument

Amperometric measurements were performed using a portable µSTAT 200 Potentiostat (Dropsens, Oviedo, Spain). Gold screen-printed electrodes (Au/SPEs DRP-220AT, Dropsens, Spain), consisting of a 4 mm smooth gold working electrode, a platinum counter electrode, a Ag reference electrode and silver electrical contacts, were used. A flow cell (DRP-FLWCL, DropSens, Oviedo, Spain) was also used for screen-printed electrodes in flow experiments. Calibration curves were fitted to a four-parameter logistic equation using Graph Prism software 9.0 (GraphPad Software, San Diego, CA, USA). LOD was calculated as the analyte concentration corresponding to 90% of the signal (IC_90_). For connecting the peristaltic pump and the flow cell a connector made of PTFE with an inner diameter of 0.5 mm was used.

#### 4.2.2. Optical Instrument

Optical measurements were performed using an OpenPlex biosensor (HORIBA Scientific, Palaiseau, France). Gold SPR sensor chips, 28.0 × 12.5 × 0.3 mm, were used, Horiba OpenPlex and EzPlex compatible, Coating: 2D carboxymethyldextran Surface. For instrument control and data analysis, OpenPlex software 2.0, SPRi-View and SPRi-Analysis, France S.A.S., France, was used. Calibration curves were fitted to a four-parameter logistic equation using Graph Prism software 9.0 (GraphPad Software, San Diego, CA, USA) and Origin Lab 8.6 (Origin Lab Corporation, Northampton, MA, USA).

### 4.3. Electrochemical Biosensor-Based Measurement

#### 4.3.1. Biofunctionalization Protocol Electrochemical Biosensor

Au screen-printed electrodes (SPEs) were cleaned using acetone, gently rinsed with water and absolute ethanol, and then air-dried until all moisture was removed. Gold surface activation was achieved through a mixed self-assembled monolayer (m-SAM), prepared by immersing the active gold surface area of the SPEs in an ethanolic solution containing 2 mM MUA for 12 h under static conditions. The chips were then gently washed with absolute ethanol, air dried and stored until use.

##### EDC/NHS Biofunctionalization

Biofunctionalization of each SPE with each of the bioconjugates (DAP-DCC-BSA, VAN-DCC-BSA, MER-DCC-BSA, COL-DMP-BSA) was performed by adding 200 μL containing EDC/NHS (10 nM in PBS) to the electrode for 1 h under static conditions, followed by 100 μL of the corresponding bioconjugate (100 μg/mL in PBS) and incubating for 3 to 4 h at room temperature. After this time, the SPEs were rinsed with PBS and the unreacted activated carboxylic acid was blocked by adding an ethanolamine solution (1 M in PBS). Finally, the biofunctionalized SPE chips were washed with water and stored at 4 °C until use [34].

##### DCC/NHS Biofunctionalization

For biofunctionalization of each SPE with each of the bioconjugates (DAP-DCC-BSA, VAN-DCC-BSA, MER-DCC-BSA, COL-DMP-BSA), 200 μL containing DCC/NHS (200 mM in DMF) was added to the electrode for 1 h under static conditions, followed by 100 μL of the corresponding bioconjugate (100 μg/mL in PBS) and incubating for 3 to 4 h at room temperature. After this time, the SPEs were rinsed with PBS and the unreacted activated carboxylic acid was blocked by adding an ethanolamine solution (1 M in PBS). Finally, the biofunctionalized SPE chips were washed with water and stored at 4 °C until use.

#### 4.3.2. Antibiotic Quantification Using Electrochemical Biosensor

A flow protocol was carried out with a flow rate of 100 μL/min throughout the procedure. The biofunctionalized SPE chip was placed in a flow cell. Prior to analysis, 1000 μL of different dilutions ranging from 1/10,000 to 1/100 of the antiserums for VAN-Rb158, DAP-Rb156, MER-Rb161 and COL-Rb 66 diluted in PBST were prepared to determine the reading conditions and parameters. On the other hand, for the calibration curve, different concentrations of the antibiotics VAN, DAP, MER and COL were prepared, ranging from 10,000 nM to 0.64 nM in 500 μL of PBST, which were mixed with 500 μL of the antibody solution for VAN-Rb158, DAP-Rb156, MER-Rb161 and COL-Rb 66 diluted 1/10,000 in PBST. The calibration curves for the electrochemical biosensor were followed by the ICH guidelines [66] for analytical methodologies.

In both cases, for the determination of the parameters and for the calibration curve, the antiserum dilutions or the antiserum/antibiotic mixture, respectively, were flowed into the sensor cell for 10 min followed by PBST buffer solution (5 min). Then, 1000 μL of the antiIgG-HRP solution (1/1000 in PBST) was allowed to flow for an additional 10 min, followed again by PBST buffer (5 min) for washing.

Citrate buffer was added to the chip for 5 min and then the chronoamperogram was started. The substrate solution was then flowed for 3 min ending the chronoamperogram. Then, the chip was washed with citrate buffer (5 min) and finally, it was regenerated by passing through VAN NaOH 0.3 M (5 min) and Glycine pH 1.5 (5 min), DAP NaCl 0.5 M (10 min), MER Glycine pH 1.5 (10 min) and COL NaOH 0.3 M (10 min) followed by PBST buffer (5 min). After regeneration, the chip was ready for subsequent analysis [34].

### 4.4. Optical Biosensor-Based Measurement

#### 4.4.1. SPRi Immobilization Protocol

Biofunctionalization protocols and calibration curve measurements were performed on a HORIBA OpenPlex, equipped with a microfluidic system and precision liquid injection. A flow rate of 5 µL/min was used for biofunctionalization and 50 µL/min for measurement protocols. For antibiotic quantification, the direct antibody/Analyte (antibiotics) strategy was used. For this purpose, three steps for analysis within optical systems were followed, (a) immobilization, (b) binding and (c) regeneration.

The sensor surfaces were previously cleaned by immersion in acetone for 1 min, followed by washing with ultrafiltered water and finally air drying until all moisture was removed. When using chips that contained a layer of 2D SCH CMDP-5 carboxymethyldextran on the surface, activation via amino coupling was used, based on covalent amino bonding, which consists of the activation of carboxyl groups with a mixture of NHS/EDC (1:1) to create N-hydroxysuccinimide esters. The flow rate of the running buffer was 5 μL/min during activation.

The immobilization solution for the antibodies was 10 mM acetate buffer, pH 4.1. The 1/1000 dilution in 10 mM acetate buffer, pH 4.1, of the antiserums was injected onto the activated surface until the level of immobilization was reached (maximum point in the association curves of the sensogram) at a flow rate of 5 μL/min. The excess of carboxyl groups that remained activated was blocked with ethanolamine hydrochloride (pH 8.5) at a flow rate of 5 μL/min. The surface concentration data are given by the equipment software when doing the analysis.

#### 4.4.2. Antibiotic Quantification Using Optical Biosensor

As mentioned above, the antibiotic quantification strategy was based on the direct interaction between antiserums for VAN-Rb157, COL-Rb 66, MER-Rb161 and DAP-Rb156 with the respective antibiotics present in the plasma samples, based on the reflectance changes where the reflected light is collected by a high-resolution CCD camera and presented as an image. The changes in light intensity are proportional to the successive molecules bound to the biosensor surface.

As with the calibration curves for the electrochemical biosensor, the ICH guidelines [67] for analytical methodologies were followed. The calibration curves were individually prepared for each antibiotic within a concentration range of 500–10,000 ng/mL. Plasma spiked with a high antibiotic concentration was prepared, followed by serial dilutions in PBST. Each curve was generated in triplicate for all concentr\ation levels.

Later, each of the dilutions was individually injected onto the sensor surface corresponding to the specific antibiotic. The injection volume was 200 μL at a flow rate of 50 μL/min. The result obtained from the binding between the antibody and the analyte was taken at the maximum point of the association of the sensograms 10 s after the injection.

After each measurement in the binding assays antigen/antibody (Ag/Ac), the chips were regenerated, ensuring that between each measurement the analyte did not interfere with the new measurements. For this purpose, the regeneration agent was previously chosen according to protocols reported in the literature based on the chemical nature of the analyte and the ligand [30,32]. After the regeneration assays, the agent chosen was 0.5 M NaCl.

#### 4.4.3. Matrix Effect of Plasma

To evaluate the matrix effect, two methodologies were carried out. For the first one, the plasma was centrifuged at 2500 rpm for 2 min and the supernatant was diluted in a range of 1/50 to 1/1000 in 0.01 M PBS, injecting each dilution individually at a flow rate of 50 μL/min, regenerating between each measurement with 200 μL of 0.5 M NaCl, at a flow rate of 50 μL/min for all antibiotic molecules. Since it was observed that there was a matrix effect by the plasma in the assay, the protocol of the second methodology reported by Tort et al. [45] was carried out. In this protocol, 100 μL of plasma was mixed with 50% (*v*/*v*) ethanol, followed by vortexing for 1 min and centrifugation at 20,000 rpm for 15 min. The supernatant was then diluted in 0.01 M PBS buffer in a range of 1/100 to 1/1000 and injected at a flow rate of 50 μL/min, regenerating between each measurement with 200 μL of 0.5 M NaCl at a flow rate of 50 μL/min. This supernatant was used to make the calibration curves [45].

### 4.5. Sensor Surface Characterization

A characterization assay of the sensor surfaces used for SPRi and electrochemical biosensors was performed. Two microscopes were used to characterize the sensor surfaces for roughness and morphology: (a) Asylum Research brand Atomic Force Microscope (AFM), model MFP-3D-BIO, using AC240TM tips with titanium and platinum coating (Oxford Instruments, Abingdon, UK) with a working frequency of 700 kilohertz and a tip radius of 28 nm ± 10 nM. (b) JEOL scanning electron microscope (SEM), model JSM 6490-LV (JEOL Ltd., Tokyo, Japan). The micrographs were taken with an acceleration voltage of 5 kV in high vacuum mode.

### 4.6. Comparison of Electrochemical and Optical Biosensor Measurements with HPLC Analysis

#### 4.6.1. HPLC Instrument

Analysis was carried out on a Hitachi LaChrom Elite HPLC system (Tokyo, Japan) equipped with an autosampler (Hitachi LaChrom Elite L-2200), a column oven (Hitachi LaChrom Elite L-2300), an HPLC pump (Hitachi LaChrom Elite L-2130) and diode array detector (Hitachi LaChrom Elite L-7455). Instrumentation control and data acquisition were achieved with OpenLAB software A.05.

#### 4.6.2. Quantification of Antibiotics Methodology

Antibiotics in plasma were quantified by HPLC using the previously standardized methodologies in the laboratory (Table 5) [66,68] and reported in the literature [69,70,71,72].

Standards were stored at 2–8 °C. Other reagents such as HPLC gradient grade acetonitrile (ACN) were purchased from Fisher Scientific (Leicestershire, UK) and ultrapure water (HPLC grade, 18.2 MU.cm) was obtained using a Milli-Q water apparatus from Millipore (Milford, MA, USA). For the mobile phase, reagents such as sodium bicarbonate, sodium hydroxide (used to adjust the pH of the mobile phase), sodium phosphate monobasic monohydrate and sodium phosphate were obtained from Sigma Aldrich (St. Louis, MO, USA).

### 4.7. Characteristics of Patient Cohorts Used for Plasma Sample Collection

Plasma samples used in the present study were obtained from previously conducted and ethically approved research projects carried out by our research group, involving patients treated with MED-222-2017 (colistin) [68], MED-317-2021 (meropenem), MED-303-2021 (daptomycin), and MED-334-2023 (vancomycin). Each of these studies received prior approval from the corresponding institutional research ethics committees, and written informed consent was obtained from all participants or their legally authorized representatives in accordance with the Declaration of Helsinki and applicable local regulations.

These cohorts were originally designed for pharmacokinetic and therapeutic drug monitoring investigations, and comprehensive demographic and clinical data were prospectively collected within those study protocols. A detailed description of the previous studies, including study design, patient selection criteria, and cohort characteristics for each antibiotic, is provided in Supplementary Table S2.

In the present study, a subset of plasma samples derived from these well-characterized cohorts was used exclusively for analytical validation and for comparison of biosensor-based quantification with the reference HPLC method. Demographic, clinical, and laboratory variables, as well as the total number of samples collected in the original cohorts, were not analyzed in the current work, as its primary objective was the analytical quantification of antibiotic concentrations in human plasma rather than clinical outcome evaluation.

## 5. Conclusions

An amperometric immunosensor has been developed for the quantification of last-line antibiotics such as colistin, vancomycin, daptomycin and meropenem in plasma. The immunosensor has good analytical characteristics and reaches favorable detection limits for the direct determination of these antibiotics in human plasma by using a gold electrode placed in a flow cell and connected to the potentiostat. Using NaOH and glycine, pH 1.5, as regeneration solutions allowed several measurements for the same chip, minimizing the differences between the data found.

The parameters of calibration curves obtained in human plasma for each of the antibiotics make this electrochemical biosensor a valuable tool for measuring colistin, vancomycin, daptomycin and meropenem levels in real time at the patient’s bedside. At the same time, the electrode surfaces presented greater roughness after immobilization and measurements due to the number of molecules that adhere to the surface between proteins, antibodies and salts.

A methodology has been designed and tested to quantify four last-line antibiotics including vancomycin, colistin, meropenem and daptomycin using an SPRi optical biosensor in human plasma, using gold chips functionalized with carboxymethyl-dextran. The biosensors showed a range of sensitivity and reproducibility that classifies them as a new methodology for the quantification of antibiotics in human plasma. This makes it a unique tool in the search for solutions to the problem of bacterial resistance.

The results of the comparison of the data obtained using the biosensors developed in this work and the reference methodology, HPLC, show that there are no significant differences between either methodology or the reference technique. This demonstrates that nanobiosensors can be used as a precise methodology for analyzing the antibiotics under study, with high reliability.

## Figures and Tables

**Table 1:** Analytical parameters of the immunosensor for COL, VAN, DAP and MER.

Antibiotic	-Imin µA	-Imax µA	Slope	IC_50_ Diluted Plasma nM (µg/L)	IC_50_ Pure Plasma nM (µg/L)	LOD Diluted Plasma nM (µg/L)	LOD Pure Plasma nM (µg/L)
VAN	0.118	1.551	−0.296	2.41 (3.49)	241 (349.2)	0.011 (0.01)	1.1 (1.59)
COL	0.2629	0.859	−1.508	4.71 (5.44)	47.11 (54.4)	0.18 (0.20)	18 (20.70)
MER	0.295	0.927	−0.538	2.14 (0.82)	214 (82.0)	0.23 (0.08)	23 (8.81)
DAP	0.336	0.448	−1.87	3.15 (5.10)	31.52 (51.06)	0.26 (0.42)	26 (42.12)

**Table 2:** Detection limits and quantification limits for antibiotics in SPRi optical biosensor (HORIBA Scientific, Palaiseau, France). Detection limits and quantification limits for antibiotics in SPRi optical biosensor.

	VAN	COL	MER	DAP
LOD	19 ng/mL	9 ng/mL	12 ng/mL	12.3 ng/mL
LOQ	28 ng/mL	21.3 ng/mL	25 ng/mL	25 ng/mL

**Table 3:** Comparison of the advantages of the electrochemical biosensor and SPR for use at point-of-care (POC) applications in hospital settings.

Parameter	Electrochemical Biosensor	SPRi Biosensor
**Time-to-result per 10 samples** **(sample preparation, measurement, and sensor regeneration)**	30 min	20 min
**Sample volume**	1000 µL	200 µL
**Cost per sample**	5 USD	10 USD
**Portability**	The unlocked biosensor is a portable device, characterized by its compact design (20 × 10 × 5 cm), light weight (200 g) and autonomous operation with rechargeable battery. Its integrated electronics allows the immediate acquisition and visualization of data in a computer, which facilitates the realization of medicines in situ without the need for additional laboratory equipment.	The biosensor is not considered portable due to its dependence on a large optical system and the need for an external power supply which limits its use to our clinicians in situ.

**Table 4:** AFM characterization studies for the electrochemical biosensor and SPRi biosensor chips. Control electrode roughness, colistin, vancomycin, daptomycin and meropenem.

	Electrochemical Biosensor			SPRi Biosensor		
**Sample**	R_a_	R_rms_	R_max_	R_a_	R_rms_	R_max_
**Control**	330.193 nm	406.336 nm	1.424 µm	1.078 nm	1.311 nm	20.142 µm
**Vancomycin**	340.720 nm	415.393 nm	1.166 µm	1.541 nm	4.751 nm	170.061 µm
**Daptomycin**	341.459 nm	414.099 nm	1.528 µm	1.146 nm	2.038 nm	62.235 µm
**Meropenem**	399.054 nm	487.112 nm	1.822 µm	1.733 nm	2.996 nm	107.434 µm
**Colistin**	406.975 nm	507.535 nm	1.483 µm	1.623 nm	2.450 nm	115.157 µm

R_a_: surface roughness, R_rms_: total root means square roughness, R_max_: maximum height difference in the plane.

**Table 5:** HPLC conditions for sample quantification by HPLC.

HPLC Conditions	VAN [69]	COL [68]	MER [70,72]	DAP [71]
Gradient	Isocratic program 88% 0.1 M phosphate buffer pH 7.0: 12% Acetonitrile	Gradient from 20 to 25% acetonitrile in 0.2 M phosphate buffer pH 6.5	Isocratic program 90% 10 mM phosphate buffer pH 7.4: 10% acetonitrile	Isocratic program 70% 0.2 M phosphate buffer pH 6.5: 30% acetonitrile
Flow rate	1.2 mL/min	1.5 mL/min		
Temperature	25 °C			
Analysis time	10 min			
Detection	UV a 240 nm	UV a 214 nm	UV a 300 nm	UV 220 nm
Column	Gemini C18, 5 μm, 150 × 4.6 mm			
Injection volume	99 µL	10 µL		
Sample treatment (Plasma)	Centrifuge 3000 rpm × 3 min. Supernatant. Dilution 1:1 in elution solvent			

## Data Availability

The original contributions presented in this study are included in the article. Further inquiries can be directed to the corresponding author.

## Supplementary Materials

The following supporting information can be downloaded at: https://www.mdpi.com/article/10.3390/antibiotics15040327/s1, Table S1: Plasma concentrations of the antibiotic’s vancomycin, colistin, meropenem, and daptomycin obtained using HPLC, electrochemical biosensors, and optical biosensors; Table S2: Characteristics of patient cohorts used for plasma sample collection; Figure S1: Surface characterization of the electrochemical and SPRi biosensors; Figure S2: Bland–Altman Correlation Analysis.
