# Supplementary material for: Advanced Biosensing Strategies for Last-Line Antibiotics Vancomycin, Colistin, Daptomycin and Meropenem: Comparative Analysis of Electrochemical and Optical Detection Methods
Source: Antibiotics (Basel). 2026 Mar 24;15(4):327. doi: 10.3390/antibiotics15040327 (PMC13113538; doi:10.3390/antibiotics15040327)
Supplement: Supplementary file 1 [file antibiotics-15-00327-s001.zip › antibiotics-4156245-supplementary/Figure S1_final.pdf]

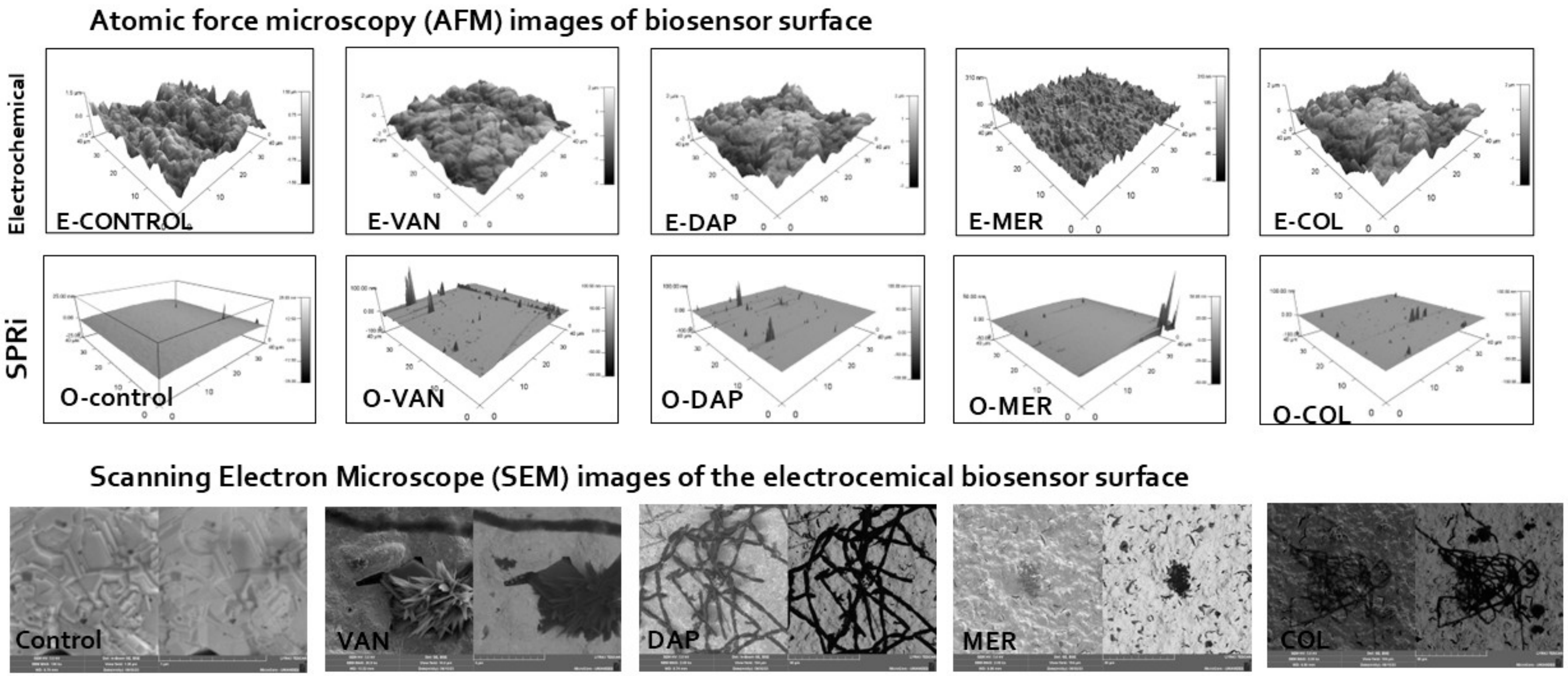

Figure S1. Surface characterization of the electrochemical and SPRi biosensors.

Electrochemical platform (E): E-Control: unmodified control chip surface (10 µm). E-VAN, E-DAP, E-MER, and E-COL: chip surfaces immobilized with antibiotic bioconjugates (10 µm), corresponding to vancomycin, daptomycin, meropenem, and colistin, respectively. Functionalized chips exhibited increased surface roughness compared with the control surface, reflecting structural modifications induced by antigen immobilization and subsequent measurements performed under constant flow conditions. SPRi platform (O): O-Control: unmodified control chip surface. O-VAN, O-DAP, O-MER, and O-COL: chip surfaces immobilized with antibiotic bioconjugates (vancomycin, daptomycin, meropenem, and colistin, respectively). A surface roughness pattern comparable to that observed in the electrochemical biosensor chips was detected. Scanning Electron Microscopy (SEM) micrographs: The images reveal the presence of organic material on the gold surface (observed as darker filamentous structures) for each antibiotic-functionalized surface as well as for the control chip. These structures may correspond to residual organic components and/or salt deposits derived from the running buffer solutions. E: Electrochemical sensor surface; O: Optical sensor surface
