# Supplementary material for: Advanced Biosensing Strategies for Last-Line Antibiotics Vancomycin, Colistin, Daptomycin and Meropenem: Comparative Analysis of Electrochemical and Optical Detection Methods
Source: Antibiotics (Basel). 2026 Mar 24;15(4):327. doi: 10.3390/antibiotics15040327 (PMC13113538; doi:10.3390/antibiotics15040327)
Supplement: Supplementary file 1 [file antibiotics-15-00327-s001.zip › antibiotics-4156245-supplementary/Figure S2_final.pdf]

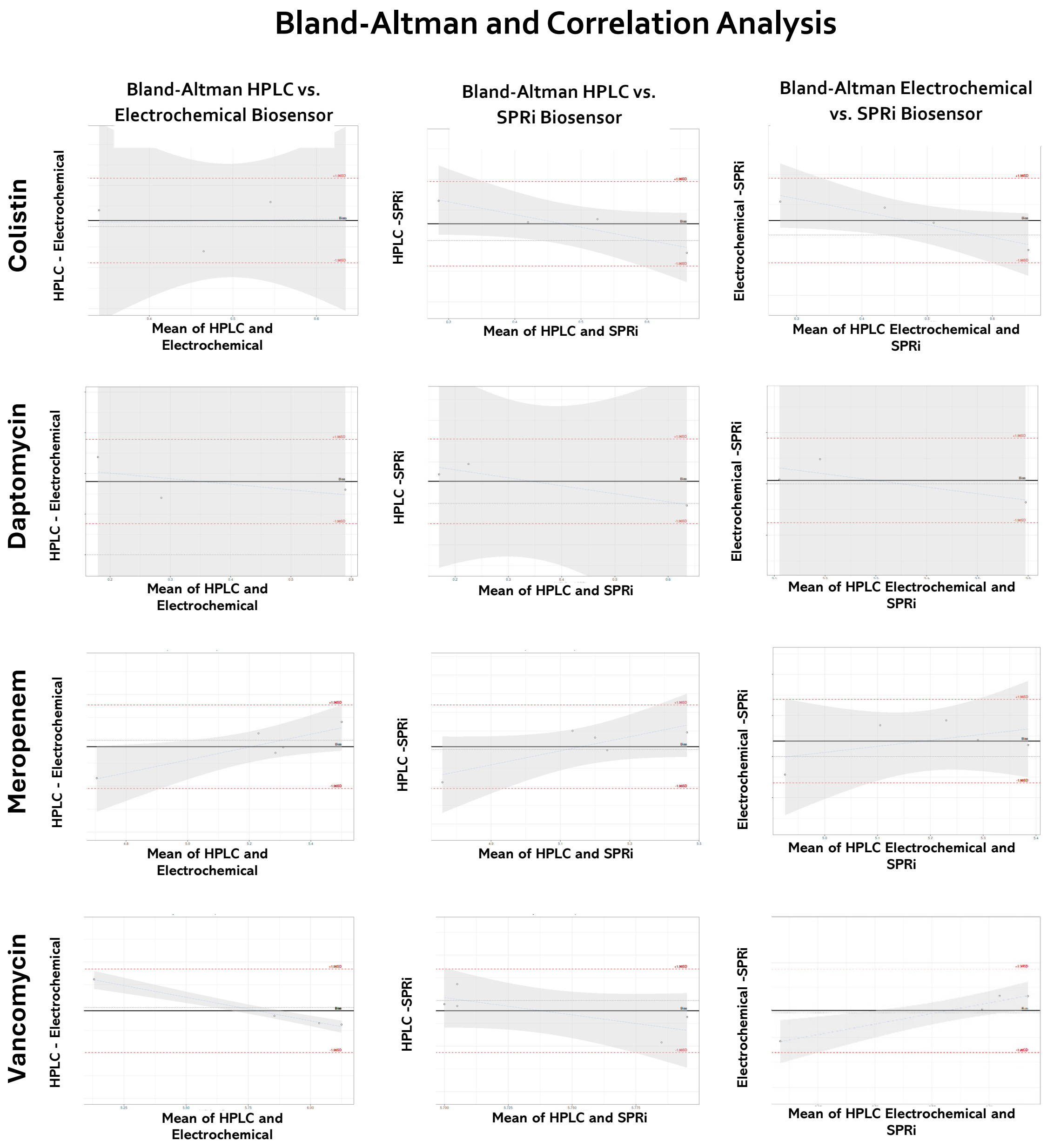

Figure S2. Bland-Altman Correlation analysis. Agreement between methods was antibiotic-dependent. Strong and statistically significant correlations were observed for colistin, daptomycin, meropenem (HPLC vs. electrochemical), and vancomycin (r ≥ 0.95 in most comparisons), generally supporting good analytical concordance. However, proportional bias was evident in SPRi comparisons (slopes > 1), and a scaling discrepancy was observed for meropenem, indicating that calibration adjustments may be required for quantitative equivalence. Overall, the results provide a rigorous assessment of analytical agreement across platforms.
