# Supplementary material for: Advanced Biosensing Strategies for Last-Line Antibiotics Vancomycin, Colistin, Daptomycin and Meropenem: Comparative Analysis of Electrochemical and Optical Detection Methods
Source: Antibiotics (Basel). 2026 Mar 24;15(4):327. doi: 10.3390/antibiotics15040327 (PMC13113538; doi:10.3390/antibiotics15040327)
Supplement: Supplementary file 1 [file antibiotics-15-00327-s001.zip › antibiotics-4156245-supplementary/Table S1.pdf]

Table S1. Plasma concentrations of the antibiotic's vancomycin, colistin, meropenem, and daptomycin obtained using HPLC, electrochemical biosensors, and optical biosensors.

Patient	HPLC (µg/mL)	Electrochemical Biosensor (µg/mL)	SPRi Biosensor (µg/mL)
		VAN	
P1	5.69		5.72
P2	5.75	4.51	5.66
P3	5.67	6.04	5.90
P4	5.75	7.08	5.84
P5	5.69	7.58	5.71
		COL	
P1	0.45	0.48	0.35
P2	0.64	0.63	0.68
P3	0.56	0.53	0.49
P4	0.35	0.33	0.22
		MER	
P1	5.23	5.34	5.24
P2	5.58	5.42	5.35
P3	4.54	4.87	4.98
P4	5.26	5.2	5.01
P5	5.28	5.34	5.12
		DAP	
P1	0.63	0.55	0.64
P2	0.32	0.25	0.13
P3	0.24	0.12	0.09

P: patient, VAN: Vancomycin, COL: Colisitin, MER: Meropenem, DAP: Daptomycin
