# Supplementary material for: Advanced Biosensing Strategies for Last-Line Antibiotics Vancomycin, Colistin, Daptomycin and Meropenem: Comparative Analysis of Electrochemical and Optical Detection Methods
Source: Antibiotics (Basel). 2026 Mar 24;15(4):327. doi: 10.3390/antibiotics15040327 (PMC13113538; doi:10.3390/antibiotics15040327)
Supplement: Supplementary file 1 [file antibiotics-15-00327-s001.zip › antibiotics-4156245-supplementary/Table S2.pdf]

Table S2. Characteristics of patient cohorts used for plasma sample collection

Parameter	Colistin (CMS) [68]	Meropenem	Vancomycin	Daptomycin
**Project Number**	MED-222-2017	MED-317-2021	MED-334-2023	MED-303-2021
**Ethics Committee Approval**	Act 12, September 2017 (Universidad de La Sabana Clinic)	Act 338, May 25, 2022 (Clínica Shaio); September 6, 2021 (Universidad de La Sabana)	Act 598, January 24, 2023 (Universidad de La Sabana)	Act 469, July 19, 2019
**Study Design**	Prospective observational	Prospective observational pharmacokinetic study	Observational cross-sectional	Prospective validation phase
**Sample Size**	Adult hospitalized patients: 57	Minimum required: 27 patients (PK design); enrolled consecutively	15 patients (minimum required: 11)	Expected 4 patients (consecutive convenience sampling)
**Inclusion Criteria**	≥18 years; hospitalized with multidrug-resistant Gram-negative infection; ≥7 days CMS treatment	≥18 years; ECMO support (VV or VA); IV meropenem; steady state achieved	≥18 years; ICU/intermediate care; clinical indication for vancomycin; informed consent	≥18 years; bacterial infection; indication for daptomycin; ≥72 h therapy
**Exclusion Criteria**	Pregnancy/lactation; prior renal failure; inadequate samples; uncontrolled infectious focus	Carbapenem allergy; renal replacement therapy; severe hepatic failure; pregnancy; no consent	Renal replacement therapy; cardiovascular surgery; ECMO; life expectancy <24 h	Incomplete antibiotic administration; CrCl <60 mL/min; cirrhosis or hepatic failure
**Sample Collection**	3 mL venous blood (EDTA) at steady state (Day 4); trough (Cmin)	Serial PK sampling after 5th, 10th, 20th doses; EDTA tubes; −80°C storage	Prior to 4th dose (steady state) and after dose adjustment; EDTA tubes	EDTA tubes; plasma separation per ISBER protocol; −20°C to −70°C storage
**Clinical Characteristics Recorded**	Age, sex, weight, infection site, Charlson index, creatinine, GFR, carbapenemase type, mortality	Age, sex, BMI, severity scores (APACHE II, SOFA), renal function, ECMO variables, microbiology	Age, sex, BMI, creatinine, GFR, comorbidities, Charlson index, dose, concomitant drugs	Age, sex, renal function, infection diagnosis
**Age**	≥18 years	≥18 years	≥18 years	≥18 years
**Sex**	Recorded	Recorded	Recorded	Recorded
**Renal Function Assessment**	Serum creatinine, GFR	Serum creatinine, Cockcroft–Gault CrCl	Creatinine, GFR	Creatinine clearance (exclusion if <60 mL/min)

P: patient, VAN: Vancomycin, COL: Colisitin, MER: Meropenem, DAP: Daptomycin
